# How to develop rapid reviews of diagnostic tests according to experts: A qualitative exploration of researcher views

**DOI:** 10.1002/cesm.12006

**Published:** 2023-04-13

**Authors:** Ingrid Arevalo‐Rodriguez, Susan Baxter, Karen R. Steingart, Andrea C. Tricco, Barbara Nussbaumer‐Streit, David Kaunelis, Pablo Alonso‐Coello, Patrick M. Bossuyt, Javier Zamora

**Affiliations:** ^1^ Clinical Biostatistics Unit Hospital Universitario Ramón y Cajal Madrid Spain; ^2^ CIBER of Epidemiology and Public Health (CIBERESP) Madrid Spain; ^3^ School of Health and Related Research (ScHARR) University of Sheffield Sheffield UK; ^4^ Honorary Research Fellow, Department of Clinical Sciences Liverpool School of Tropical Medicine Liverpool UK; ^5^ Li Ka Shing Knowledge Institute, St. Michael's Hospital Unity Health Toronto Toronto Ontario Canada; ^6^ Epidemiology Division and Institute for Health Policy, Management, and Evaluation, Dalla Lana School of Public Health University of Toronto Toronto Canada; ^7^ Cochrane Austria, Department for Evidence‐based Medicine and Evaluation University of Krems Krems Austria; ^8^ Canadian Agency for Drugs and Technologies in Health (CADTH) Ottawa Ontario Canada; ^9^ Iberoamerican Cochrane Center‐Servicio de Epidemiología Clínica y Salud Pública Biomedical Research Institute (IIB‐Sant Pau) Barcelona Spain; ^10^ CIBER of Epidemiology and Public Health (CIBERESP) Barcelona Spain; ^11^ Department of Epidemiology & Data Science, Amsterdam Public Health Amsterdam University Medical Centres Amsterdam The Netherlands

**Keywords:** accelerated methods, diagnostic tests, evidence synthesis, rapid reviews, systematic reviews

## Abstract

**Background:**

Rapid reviews (RRs) have been used to provide timely evidence for policymakers, health providers, and the public in several healthcare scenarios, most recently during the coronavirus disease 2019 pandemic. Despite the essential role of diagnosis in clinical management, data about how to perform RRs of diagnostic tests are scarce. We aimed to explore the views and perceptions of experts in evidence synthesis and diagnostic evidence about the value of methods used to accelerate the review process.

**Methods:**

We performed semistructured interviews with a purposive sample of experts in evidence synthesis and diagnostic evidence. We carried out the interviews in English between July and December 2021. Initial reading and coding of the transcripts were performed using NVIVO qualitative data analysis software.

**Results:**

Of a total of 23 invited experts, 16 (70%) responded. We interviewed all 16 participants representing key roles in evidence synthesis. We identified 14 recurring themes including the review question, characteristics of the review team, and use of automation, as the topics with the highest number of quotes. Some participants considered several methodological “shortcuts” to be ineffective or risky, such as automating quality appraisal, using only one reviewer for diagnostic data extraction and only performing descriptive analysis. The introduction of limits might depend on whether the test being assessed is a new test, the availability of alternative tests, the needs of providers and patients, and the availability of high‐quality systematic reviews.

**Conclusions:**

Our findings suggest that organizational strategies (e.g., defining the review question, availability of a highly experienced team) may have a role in conducting RRs of diagnostic tests. Several methodological shortcuts were considered inadequate for accelerating the review process, though they need to be assessed in well‐designed studies. Improved reporting of RRs would support evidence‐based decision‐making and help users of RRs understand their limitations.

## 1 BACKGROUND

Rapid reviews (RRs) have emerged as a potentially efficient alternative to resource‐intensive systematic reviews (SRs) performed within time constraints [1]. Although currently there is no consensus on what defines a RR, it is commonly accepted that this approach aims to speed up the review process by implementing diverse methods and strategies, such as narrowing the review question and introducing automation for database searching and study selection [1, 2, 3]. Over the past decade, RRs have been used to provide timely evidence for decision‐making in various contexts [4, 5, 6], most recently for the coronavirus disease 2019 (COVID‐19) pandemic [7].

Diagnostic tests are essential in guiding healthcare management. While the methods for performing SRs of test accuracy studies are well‐established [8], data are scarce about how to perform RRs of diagnostic tests, which presents particular challenges given the unique methods used to assess methodological quality and implement the statistical methods for test accuracy studies [8, 9, 10, 11]. In a previous scoping review, we examined the characteristics of RRs of diagnostic tests by searching for reports in repositories of Health Technology Assessment agencies and in multiple databases to July 2019 [12]. We found most diagnostic RRs were broad in scope and assessed multiple index tests, test applications, and outcomes. We also found that well‐known RR strategies, such as setting limits for literature searching by date, language, or the number of databases, were rarely reported [12]. In an international survey among representatives of institutions performing diagnostic evidence syntheses, participants reported a greater number of methodological shortcuts than those identified in the scoping review. In addition, we found that RR methods are usually applied to diagnostic appraisal without knowledge about their potential effects on review quality [13].

In this study, we explored the views and perceptions of experts in evidence syntheses and diagnostic evidence about the utility, value, and effect of methods considered to accelerate the review process. We followed a previously published protocol [14]. We used qualitative methods to collect data on the views and perceptions of researchers conducting evidence syntheses of diagnostic tests. We wanted to explore in‐depth how, why, and when methods labelled as “rapid” were used and their effect on quality. Researchers worldwide are increasingly using RR methods [15], so we were keen that this study involves researchers from diverse countries and geographical regions, to identify, if possible, any country‐specific views and practices that might be transferable elsewhere [2].

## 2 METHODS

The following outline of the guidance draws on the Consolidated Criteria for Reporting Qualitative Research (COREQ) [16] and the Standards for Reporting Qualitative Research (SRQR) [17]. In accordance with the Spanish National Regulation, this study has been exempted from approval by the Institutional Ethics committee for Investigation because the research only involved professionals in the field and not patients.

### 2.1 Design

We performed online semistructured interviews with a purposive sample of experts and users of diagnostic evidence syntheses. We carried out the interviews between July and December 2021. The sampling strategy allowed us to select people who were diverse according to geographical region, country, organization, and role. The key roles we sought were methodologists, biostatisticians, information specialists, developers of RRs guidance, and knowledge users (also called stakeholders). Using a list of relevant organizations and roles developed by the study authors and results of previous studies [12, 13], we identified 23 experts in evidence synthesis. We invited all 23 potential interviewees by email and sent one reminder to people who did not respond.

In this study, we defined RR as a knowledge synthesis strategy using limited or accelerated methods to expedite the time required to obtain a conclusive answer [18]. Also, we defined a diagnostic test as any method for collecting additional information about a patient's current or future health status. Diagnostic tests include symptoms and signs, physical examination, and laboratory and imaging strategies [19].

### 2.2 Data collection methods and instruments

We based the interview questions on findings from a prior scoping review and international survey [12, 13]. We developed an interview guide and, following input from the study team, further refined the guide (see Supporting Information: Material 1). Before data collection, the lead study author (I. A. R.) and another author (K. R. S.) carried out a pilot interview to test the questions, interview method, and transcription. After receiving informed consent, I. A. R. conducted interviews using an online platform (Zoom Video Communications Inc.), with audio and video in use during the interview. Interviews lasted 30–45 min. We used a semistructured approach whereby we explored comments from participants if they related to other relevant issues. We held interviews in English, as all participants were familiar with English. We recorded interviews using the online platform recording function. The recordings were initially processed by transcription software (Sonix Inc.) to produce a word document from each interview for qualitative analysis. The interviewer read through the documents to check for content and formatting errors in transcription before sending them to another study author (S. B.), an English speaker, responsible for data analysis.

### 2.3 Data processing and analysis

We initially developed a coding framework based on findings from a literature review [12]. We then iteratively refined the codes using thematic analysis. We used NVIVO qualitative data analysis software Version X (Lumivero Inc.) for coding the transcripts. Each transcript was read line by line, and segments of text relating to a particular idea or theme were assigned a code by the researcher for data analysis. Then, existing themes were combined or new themes established. Subsequently, the coding framework and list of quotes assigned to each code were returned to the interviewer. To identify differences in opinion regarding the coding, the interviewer and researcher for data analysis met online to discuss data interpretation further. The interviewer agreed that the main themes identified represented the key discussion areas reported by participants during the interviews.

### 2.4 Trustworthiness

The sequential nature of the interviews allowed us to confirm and explore topics with participants who were interviewed later. The software used for analysis indicated where themes were developed from comments by a few or many participants. During the analysis, we highlighted instances where participants had different viewpoints. A study author (S. B.) independently coded the data to ensure consensus between the study authors. The interviewer confirmed that the analysis accurately represented their recollections.

## 3 RESULTS

From the list of 23 potential participants, 16 experts (70)% replied to our email, and all agreed to be interviewed. Participants included eight SR authors, two information specialists, two experts producing RR guidance, three biostatisticians, and one knowledge user based in a European agency for healthcare products. Of the total 16 participants, 11 (69%) were women and 10 (62%) had published five or more SRs of test accuracy during the last 10 years. Participants were based in Europe (Belgium, Germany, Ireland, the Netherlands, Sweden; *n* = 9), the United Kingdom (*n* = 5), and America (Canada and the USA; *n* = 2). All participants were involved in developing or using evidence syntheses of diagnostic tests before and during the COVID‐19 pandemic, usually with important time constraints and the use of various review strategies and shortcuts. We identified 14 recurring themes related to the performance of RRs of diagnostic tests (Table 1).

**Table 1:** List of themes and frequency‐semistructured interviews about RRs of diagnostic tests.

Theme	Number of interviews providing a quote	Total number of quotes
General views about RRs and their role	15	46
Research question and scope	16	77
Characteristics and skills of the review team	15	71
Use of automation and review software	15	66
Screening and data extraction	15	55
Assessment of certainty of the evidence and quality appraisal	16	53
Limiting searches/general	11	36
Role of knowledge users/evidence users	11	35
Parallel work/carrying out tasks in parallel	10	29
Use of existing systematic reviews	13	23
Language limitations	10	16
Limiting outcomes	8	13
Synthesis approach	6	10
Date limitations	5	7

Abbreviation: RR, rapid review.

### 3.1 General views about RRs and their role

**Table:** 

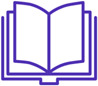	**Representative quotes**
	I think it's really context‐dependent. How much time do you have? What is the question? How broad is the scope? And that can inform the decisions that you make about the other methods. (Developer of RR guidance #1]
	I think it's a balancing act between the accelerated methods, the timeframe you have available and the confidence and certainty in the conclusions of the review. (Developer of RR guidance #2)
	It's a little bit unreasonable to assume you'll complete a full systematic review in a short amount of time just doing things quicker. (Information Specialist #2)

Participants expressed their general views about expediting diagnostic reviews by drawing on the methods usually associated with RRs. Most considered these methods acceptable but highlighted that clearly defining the scope of the review and review question(s) were crucial. Interviewees described the need for “a balancing act,” weighing time and resources against the potential effect on quality. In addition, Interviewees acknowledged that it was not fully understood how methods to increase the rapidity of the review process affected the quality and conclusions of RRs, and there was a need for research regarding the effects of these methods.

While one participant reported “mixed feelings” and a perception of “cutting corners” with RRs, others highlighted their advantage over SRs, in particular, if an SR took a long time, the findings could be outdated. Participants emphasized that carefully considering the topic and purpose of RRs was necessary. It was also important to report methods to increase rapidity if used.

### 3.2 Research questions and scope

**Table:** 

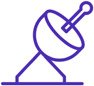	**Representative quotes**
	The starting point is to try to answer something focused and not broad. So I think that would be key. (Methodologist #5)
	I found it useful limiting the number of potential applications of the test…limiting the number of index steps to assess only in context and setting in use, and also limiting the population to determine the exact intended population and not any other population. (Knowledge user #1)
	It depends on the clinical pathway of the target condition; if, for example, you already know that children are not really infected, then you can start limiting the population. But if you don't know that, I would not limit. So again, I think whether they are unacceptable or not depends on the situation. (Methodologist #7)

All participants emphasized that the first consideration for carrying out a review quickly was developing the scope and review question(s). Interviewees highlighted the need for “focused,” “specific,” “well‐defined,” and “do‐able” research questions as “everything follows from that.” Limiting the review question by population and context was considered acceptable for RRs of diagnostic evidence, although one participant emphasized that this depended on what was already known. Others agreed that the appropriateness of limiting the review question also relied on the topic and situation, for example, how many tests were available, key clinical needs, and the urgency of the review question.

### 3.3 Characteristics of the review team

**Table:** 

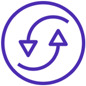	**Representative quotes**
	The team, to me, is the critical factor, and one of the most critical factors in us being able to accelerate. (Developer of RR guidance #1).
	Recruiting really skilled people in all steps, that's great. Usually, if people know exactly what they're supposed to do and everything is clear, none of these steps has to take that long. (Methodologist #4)
	I think there's a key role related to managing the projects. I think we can't underestimate how intense that position is, especially in a rapid review and trying to organise multiple people in a short amount of time, plus taking on some burden of systematically reviewing yourself usually. (Methodologist #1)

Interviewees emphasized that a highly experienced team was required if diagnostic reviews were to be performed rapidly. Participants also noted the need for adequate staffing, capacity, and readiness to begin work on a project. In addition, participants described how expertise from clinical colleagues had an important part to play, mainly if a review question was complex or the research identified was challenging to interpret. Good team coordination was noted to be a vital part of RRs, with regular communication between team members and management.

Participants suggested identifying a person to coordinate all stages of RRs. The coordinator could assign staff to work on different parts of the review as needed, draw on the strengths of individual reviewers, and quickly resolve queries and inconsistencies. Interviewees recommended supporting junior team members by using a “buddying up” system where more and less experienced staff worked together. The requirement for training was also highlighted. All participants emphasized that a large team of experienced staff requires adequate resources.

### 3.4 Use of automation

**Table:** 

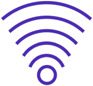	**Representative quotes**
	I definitely think they can play a role in the screening and the selection of references. I'm very doubtful about using it in quality appraisal. You can highlight some keywords, of course. But the problem is more of interpretation. (Methodologist #5)
	The good ones pop up first. You select them for full text, and the full‐text team can already start quite rapidly. (Methodologist #7)
	It cannot be automated; maybe it can be semi‐automated in the future, but definitely not fully automated. (Methodologist #3)

There were varying levels of knowledge and experience in using software or automated systems to assist with RRs (i.e., researchers with only general knowledge, researchers with only practical experience, and experts involved in tailoring and developing software). It was perceived as “just a matter of time” before these tools were utilized more fully. Participants viewed the screening and study selection process as the step where automated systems could be of most value owing to the large number of records often identified when searching for test accuracy studies. There was less confidence that these techniques could be used successfully for data extraction or quality assessment, such as an automated QUADAS‐2 tool. Those who had used automated systems to expedite screening suggested that they were a valuable “second checking system” or could be used as part of a prioritization process by highlighting studies that had a greater chance of being relevant. Participants highlighted challenges regarding automated methods still in development, noting that diagnostic studies are often poorly reported, which would adversely affect software capabilities, and that diagnostic testing research offers particular challenges. While generally supportive of automated tools, some interviewees emphasized the need to evaluate the effectiveness of artificial intelligence and related technologies. Some referred to “semiautomation,” stressing that human involvement was still needed.

### 3.5 Screening and data extraction

**Table:** 

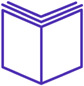	**Representative quotes**
	I think to have only one data extractor, no matter how experienced that person is, is problematic. (Methodologist #4)
	I don't know really whether it's acceptable, but I'm not a big fan of only one reviewer. And because sometimes, especially in the field of diagnostics, it's more difficult. It's something more difficult to see what the exact patient population is, which can differ a lot within the diagnostic field. (Information Specialist #2)
	You can also choose a middle route so that the data extraction is done by only one reviewer but checked by another person independently. (Methodologist #1)

Most participants considered using only one person to assess methodological quality and extract data as being “problematic.” However, this strategy was considered acceptable for screening records by title and abstract to remove “the obvious noise.” The main reason reported by participants to avoid using only one person in other stages of the review was the possibility of incorrectly recording critical characteristics, such as the population of interest, the positivity criteria of the index test, and the data needed for statistical analysis (e.g., prevalence of the target condition, number of false positives).

An approach suggested as “a middle route” involves using two reviewers for the assessment of methodological quality and data extraction, one reviewer to provide judgments about quality and applicability and extract data and a second reviewer to check this information (selective verification). However, participants noted it would be desirable to establish rules about what to do if the second reviewer found errors (e.g., check a random sample of studies). Participants also suggested that someone from the core team be involved in these stages of RRs to support the process.

### 3.6 Assessment of certainty of the evidence and quality appraisal

**Table:** 

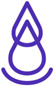	**Representative quotes**
	You can't skip quality assessment; you do have to critically appraise the studies, so that one is an essential step. (Statistician #1)
	I don't think it should just be down to a single person. I just know how error‐prone those activities are. (Information Specialist #1)
	If you've got four people doing a quality appraisal, then the second reviewer should always be the same person. (Methodologist #7)
	You could consider limiting your review to the highest quality studies. So, for example, exclude the case‐control studies for diagnostic tests. (Methodologist #4)
	I think GRADE is completely so needed in diagnostic rapid reviews. It's the way that we communicate the findings to people who aren't familiar with the synthesis methods and understand them. (Methodologist #5)
	I think not having a GRADE assessment would not really impair your review. I mean, it's helpful, but I don't think a review becomes less trustworthy if you don't. (Methodologist #6)

There was consensus among participants that assessment of methodological quality using tools such as QUADAS‐2 was essential and should be retained even if there were only a short time to perform the review. Participants agreed that more than one reviewer should be involved in the quality appraisal, although for some participants, it was adequate to have a sample of the appraisals checked by a second reviewer. Few participants viewed having a single reviewer for quality assessment as an acceptable way to increase the rapidity of the review process, and, for them, it was best avoided. However, participants thought that having too many team members perform quality assessments could lead to consistency problems. A solution suggested for situations involving more than two reviewers was to have a “core” person overseeing all appraisals. One participant suggested that methodological quality assessment could be done more quickly by selecting particular items from a standardized tool. Another option would be to restrict the inclusion criteria so that only higher‐quality studies were eligible for the review.

While participants agreed that assessment of methodological quality should not be omitted from a review to enhance rapidity, there was less consensus regarding the assessment of the certainty of the evidence, such as the GRADE (Grading of Recommendations Assessment, Development and Evaluation) approach.

Eight participants (50%) considered assessment of the overall certainty to be an essential way of presenting the evidence, while the other eight participants disagreed and viewed methodological quality assessment as being sufficient.

### 3.7 Limiting searches/general

**Table:** 

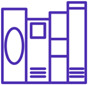	**Representative quotes**
	It's not so much about the number of sources you search. It's about making sure you've got the right types of sources. (Information Specialist #2)
	There is no perfect process anyway, and if you're trying to do something, then under time constraints, potential compromises have to be made…to apply some of these things to the search…may mean that the odd study is missed, but that on the whole, I would be very surprised if this dramatically affected the conclusions of the review. (Information Specialist #1)
	You need to think carefully about the search and getting the balance right between being comprehensive, getting your strategy right, but thinking about your resources. (Methodologist #2)

Views differed regarding expediting the review process by limiting the number of electronic databases to be searched. Some participants emphasised that the focus should be on identifying the right sources, while others thought several databases should always be searched. Participants recognized that methods to limit database searching would depend on the topic and research questions. Opinion was divided on methodological filters to reduce the number of records retrieved for screening. Some respondents advocated their use, suggesting the need for a pragmatic approach, whereas others opposed the use of filters, considering them as “not being very accurate” or “risky.” Participants also commented about the need to be pragmatic when searching other sources, such as grey literature and conference abstracts, acknowledging that decisions were often made on a topic‐by‐topic basis, and depended on time and available resources.

### 3.8 Role of knowledge users/evidence users

**Table:** 

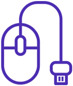	**Representative quotes**
	So I think the main part of this is having a dialogue with the target audience or those needing this answer to make sure that we really think this is the most important part when doing something rapidly to really define what is exactly we need to answer here. (Developer of RR guidance #2)
	I think we should be telling the Commissioners of the reviews, here's the product you're going to get, here's the time frame you can get it in, so here's the question which meets your needs. We can do it this way or this way. (Methodologist #8)
	You choose the most relevant one for the clinical practice. So it's not always essential that you are as complete as possible, but sometimes you just need to include the bottlenecks in practice or the bottlenecks in policymaking. (Methodologist #3)

All participants emphasized how knowledge users' requirements and wishes influenced the planning and conduct of RRs. Knowledge users' engagement was critical to defining the diagnostic question and ensuring the review was relevant to evidence users. The need for debate to ascertain how urgently an answer was needed was described, with collaborative discussion required to agree on the scope and focus of the RR. The role of knowledge users was reported to be particularly important when there were options regarding where to focus within the limited time.

### 3.9 Parallel work

**Table:** 

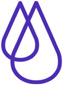	**Representative quotes**
	I think that should be definitely considered using multiple reviewers in parallel and overlapping is probably the key thing to increasing the speed in a lot of these. (Methodologist #1)
	I think it's about making sure you've got that robustness and that consistency across all of those reviews so they do it in the same way. (Statistician #4)

Carrying out tasks in parallel rather than sequentially was recommended to increase the rapidity of performing the reviews. Participants shared several examples of parallel working: some reviewers screen abstracts while others look at potentially eligible full texts; some reviewers screen for eligibility while others extract data; some reviewers assess quality while others extract data. An example of using international teams from different time zones was described, enabling work to be continued in parallel across 24‐h periods.

While the benefits of parallel working were emphasised, several potential challenges were also mentioned, including the need to have a large team available, for the team members to be experienced and familiar with working together, for there to be brief regularly scheduled meetings, and for the project to be managed well with one person coordinating and supervising. One participant commented on the need for a pilot phase, and others emphasized the need to ensure consistency between reviewers.

### 3.10 Use of previous reviews

**Table:** 

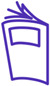	**Representative quotes**
	I think using a previous review as a starting point is, in theory, a really good idea. But of course, it depends on the methods that have been used for that previous review and feeling confident that you can rely on its findings. (Information Specialist #1)
	I don't think that you can take a previous review, only perhaps as a source of studies. I think you would still have to go back to the studies that were in it. I have hesitations about using a previous review because I think you're always going to want to dig in a bit more to the studies that were in it. (Methodologist #2)

There was consensus amongst participants that identifying existing SRs was valuable to the RR process, with the caveat that the quality of a previous diagnostic SR needed to be considered. It was suggested that RRs could be restricted to updating a previous SR if an existing high‐quality review was identified. One methodologist, however, commented that this was not necessarily quicker or cheaper, and another held the view that previous reviews should only be used as a source of studies.

### 3.11 Language limitations

**Table:** 

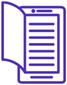	**Representative quotes**
	At the moment, we have so many easy tools for translation that I really don't see the problem anymore of including as many languages as possible. So I wouldn't recommend this. I don't think it helps a lot. (Methodologist #4)

There was considerable debate concerning language limitations in RRs (typically limited to sources in English). While some respondents reported filtering for English publications (describing time and cost reasons), others considered that “it's wrong to be limited by language,” citing the availability of online translation tools, use of an international team, and potential biases from excluding studies carried out in non‐English‐speaking countries.

### 3.12 Limiting outcomes

**Table:** 

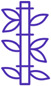	**Representative quotes**
	When it comes to sort of following, for example, patient‐important outcomes and so on, it can be important to make sure to consider that you really are addressing the patient‐important outcomes that are relevant for this patient group and not just listen to those asking the question that might not have the full picture. (Statistician #1)
	I would definitely limit the number of potential applications, but also the number of outcomes and actually, if you think about a rapid review on a diagnostic test, then for me the most. A useful outcome would be just simply diagnostic accuracy. And then if that sort of is promising, then you can move on to the next stage where you would either do a full, not rapid review or you would expand to other outcomes such as costs or impact of the test. But I guess for a rapid review, and a diagnostic test is limited to diagnostic accuracy. (Methodologist #6)

Regarding increasing the rapidity of reviews by limiting outcomes, participants emphasized the importance of outcomes being relevant to the research question and of importance to patients, clinicians and policymakers. However, there was also recognition that limiting outcomes (e.g., including only sensitivity and specificity) might make reporting the review less generic, “more concise, and easier to analyze and interpret the findings.” Some participants described how poor quality of primary studies meant that important outcomes were not reported, and therefore “any outcomes reported should be considered.”

### 3.13 Synthesis approach

**Table:** 

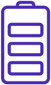	**Representative quotes**
	If you've got quite a lot of different thresholds or heterogeneity and you're trying to communicate, they're trying to do that by only doing a narrative synthesis would be really, really hard. I think by having a ROC curve in, there would be so much more helpful. (Statistician #2)

Potential limitations of carrying out a narrative synthesis were described, with participants viewing statistical synthesis as preferable except when few primary studies were available. One participant described the potential for narrative synthesis to become “expert opinion,” and statistical synthesis was described as a better method for communicating findings of reviews of test accuracy. Having input from clinicians to interpret results was recommended, together with providing a complete description of the included studies.

### 3.14 Date limitations

**Table:** 

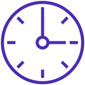	**Representative quotes**
	The tests you're interested in only became commercially available after a certain date. So, of course, there's no point searching from the beginning of time when you know that it's only been available in the last five years. (Methodologist #3)

There was consensus that limiting searches by date was an appropriate way for RRs of diagnostic tests. This strategy was beneficial, in particular, when newer versions of a test were available.

## 4 DISCUSSION

To the best of our knowledge, this is the first qualitative study of the views and perceptions of experts in evidence synthesis and diagnostic evidence about how, why, and when to use RRs methods, as well as the effects on review efficiency and quality. We used a purposive sampling strategy to identify experts representing different roles during the development and use of diagnostic evidence syntheses. Hence, we obtained multiple points of view about these methods and their use in developing diagnostic RRs. From this group of experts, we identified 14 recurrent themes related to the conduct of RRs of diagnostic tests, including the review question, characteristics of the review team, and use of automation as the topics that received the most quotes.

We found that participants expressed mixed opinions about the relevance and use of RRs in diagnostic tests but emphasized that clear reporting would help readers understand the limitations of evidence syntheses in this field. Although there is currently no PRISMA extension for RRs, the PRISMA checklist for SRs has been recommended as a reporting template while a tailored checklist for RRs is developed [20, 21]. We also found that organizational strategies for RRs, such as the need for careful planning of the review question and the characteristics of the review team, were frequently highlighted. The critical role of these strategies has been emphasised previously by Rehfuess et al. [22] as a lesson learnt from developing RRs on public health during the COVID‐19 pandemic. In addition, Tricco et al. [7] also found that a team with adequate skills and collaborative working arrangements (e.g., including knowledge users in review planning and development) have a role when operating in a short timescale. Scoping of the review question would benefit from knowledge users' input to plan further steps [23, 24, 25]. Although these strategies are not methodological shortcuts, we agree they could be valuable for both SRs and RRs of diagnostic tests in the current practice [26].

Some of the RR methods evaluated were considered inadequate or risky, such as the use of automation for quality assessment, involvement of only one reviewer for data extraction, and default use of descriptive analysis (in lieu of statistical analysis). Introduction of limits (i.e., in the scope of the review, the number of databases searched, and the date of searching) might depend on what is already known about the diagnostic test if the tool under assessment was recently developed, clinical needs informed by knowledge users, the presence of alternative tests, and the availability of high‐quality SRs. In addition, some of the strategies suggested, such as omitting assessment of the certainty of evidence and statistical analysis, might have little impact on speeding the review process (i.e., they are not essentially time‐consuming), although they reflect the need to include experts with adequate skills on the review team.

We acknowledge several potential limitations of our study. We planned to sample a larger number of participants to include a wide range of opinions about the challenges of using RR methods and to compare practices and perceptions among countries. Although our final sample included participants covering all preplanned roles involved in developing and using diagnostic evidence syntheses, the limited number of participants could have resulted in selection bias. Data analysis indicated evidence of saturation of themes, although this may result from a limited pool of respondents. In addition, the study author who performed the interview is also a methodologist involved in diagnostic evidence synthesis, and her interpretations could be biased. We also recognize that our interviewees' opinions were primarily based on their experiences synthesizing evidence about tests for diagnosing severe acute respiratory syndrome coronavirus 2. However, while the role of RRs in informing clinical practice was not fully established before 2020, the COVID‐19 pandemic underscored the need for rapid evidence syntheses to help manage healthcare emergencies and the need for standard methods for their conduction [15, 27, 28, 29].

Regarding future research, participants stressed the need for empirical assessment of these methods in the diagnostic field. A scoping review of studies that had appraised methodological shortcuts for performing RRs identified few studies that had formally evaluated these methods [30]. Recently, there have been efforts to assess standard RR methods for diagnostic evidence [31]. The evaluation of strategies for the selection of records of diagnostic studies (e.g., using one reviewer to discard irrelevant titles), as well as strategies for complex activities (e.g., quality assessment and data extraction using skilled researchers who perform these tasks simultaneously), might provide valuable information to researchers developing diagnostic evidence syntheses.

## 5 CONCLUSIONS

Our findings suggest that organizational strategies (e.g., availability of a highly experienced team) may have a role in conducting RRs of diagnostic tests. Some methodological shortcuts were considered inadequate or risky in speeding the review process, although they need to be assessed in well‐designed empirical studies. Clear reporting of RRs would support evidence‐based decision‐making and help users of RRs understand their limitations.

## AUTHOR CONTRIBUTIONS

**Ingrid Arevalo‐Rodriguez, Karen R. Steingart, Andrea C. Tricco, Barbara Nussbaumer‐Streit, David Kaunelis, Pablo Alonso‐Coello, Susan Baxter, Patrick M. Bossuyt, and Javier Zamora**: Conceptualization; design of the study. **Ingrid Arevalo‐Rodriguez, Karen R. Steingart, Andrea C. Tricco, Barbara Nussbaumer‐Streit, David Kaunelis, Pablo Alonso‐Coello, Susan Baxter, Patrick M. Bossuyt, and Javier Zamora**: Protocol development. **Ingrid Arevalo‐Rodriguez**: Coordination. **Ingrid Arevalo‐Rodriguez, Andrea C. Tricco, Javier Zamora, and Barbara Nussbaumer‐Streit**: Information collection; data analysis. **Ingrid Arevalo‐Rodriguez, Karen R. Steingart, Andrea C. Tricco, Barbara Nussbaumer‐Streit, David Kaunelis, Pablo Alonso‐Coello, Susan Baxter, Patrick M. Bossuyt, and Javier Zamora**: Writing—original draft. **Ingrid Arevalo‐Rodriguez, Karen R. Steingart, Andrea C. Tricco, Barbara Nussbaumer‐Streit, David Kaunelis, Pablo Alonso‐Coello, Susan Baxter, Patrick M. Bossuyt, and Javier Zamora**: Read and approved the final manuscript.

## CONFLICT OF INTEREST STATEMENT

The authors declare no conflict of interest.

## ETHICS STATEMENT

Following the Spanish National Regulation, this study has been exempted from approval by our Ethics committee for Investigation (Hospital Ramon y Cajal, communication received on November 6, 2018).

## Supporting information

Supporting information.

## Data Availability

The data sets used and/or analyzed during the current study are available from the corresponding author upon reasonable request.
