# Supplementary material for: How to develop rapid reviews of diagnostic tests according to experts: A qualitative exploration of researcher views
Source: Cochrane Evid Synth Methods. 2023 Apr 13;1(2):e12006. doi: 10.1002/cesm.12006 (PMC11795928; doi:10.1002/cesm.12006)
Supplement: Supplementary file 1 — Supporting information. [file CESM-1-e12006-s001.pdf]

Challenges of rapid reviews for diagnostic test questions: qualitative stage

instituto ramón y cajal de investigación sanitaria irycis

ciberesp
Centro de Investigación Biomédica en Red
Epidemiología y Salud Pública

MINISTERIO DE CIENCIA, INNOVACIÓN Y UNIVERSIDADES

Instituto de Salud Carlos III

FONDO EUROPEO DE DESARROLLO REGIONAL
Una manera de hacer Europa
FONDO SOCIAL EUROPEO
El FSE invierte en tu futuro
Unión Europea

Background

- We developed and administered an online questionnaire seeking information about the methods and resources involved in the performance of RRs of diagnostic tests.
- On April 2019, we invited representatives from institutions around the world who perform evidence syntheses to participate in this closed survey (n=25)

Table 1 Characteristics of participating institutions

Country	N (%)
Africa	1 (4)
America	9 (36)
Asia	3 (12)
Europe	11 (44)
Oceania	1 (4)
**Number of RRs of diagnostic tests developed**	
Less than 10 RR	10 (40)
10 to 30 RR	9 (36)
More than 30 RR	6 (24)
**Availability of RR methodological guidance (i.e. handbook)**	
Yes	9 (36)
**Structure of RRs team**	
High level of training	22 (88)
Involvement of more than two reviewers or more than one team	10 (40)
Stakeholder involvement in several activities	10 (40)

https://doi.org/10.1186/s12874-020-01004-z

Definitions

- In our research, we defined **rapid review (RR)** as a knowledge synthesis strategy using limited or accelerated methods to expedite the time required to obtain a conclusive answer (time-constraints).
- Also, we defined a **diagnostic test** as any method for collecting additional information about the current or future health status of a patient. Diagnostic tests include symptoms and signs, physical examination, laboratory and imaging strategies.
- For the purpose of this interview, we divided the methods used by developers into four groups (see following slides). Methods are presented by frequency of reporting

Consent to participate and to record the interview

<u>A. Scope of the review:</u>

Those methods helping to define the scope of the RR, including those limiting the number of populations, interventions and outcomes considered

Method	%
**Defining a structured question**	92
**Limiting the number of potential applications of the tests**	80
**Limiting the number of index tests**	76
**Discussing the clinical pathway for the target condition**	68
**Limiting the population**	68
**Limiting the number of comparisons**	56
**Limiting the number of outcomes**	44
**Limiting the accepted reference standards**	4

Key questions
Which of these methods for defining the scope of a review would you recommend (consider) when defining the scope of a Diagnostic RR? Why?
Are any of these methods unacceptable for a high quality RR? If so why?
Would you implement any of these methods for defining the scope of a Diagnostic RR? Why/why not?

B. Implementation of review shortcuts:

When one or more systematic review steps may be reduced or omitted

Method	%
Using a previous review as a starting point	92
Excluding additional searches	88
Limiting search strategies by language	84
Performing a narrative synthesis of findings	76
Limiting search strategies by date	68
Limiting the data abstraction: one reviewer only	64
Limiting the selection of full texts: one reviewer only	60
Excluding a GRADE assessment of findings	60
Limiting search strategies results using methodological filters	56
Limiting screening of titles & abstracts: one reviewer only	48
Limiting the quality appraisal: one reviewer only	40
Limiting the syntax of search strategies	32
Limiting search strategies to one database	8
Selecting pre-existing synthesis of evidence only	4
Selected verification for screening, final selection, data extraction, quality appraisal	4

Key questions
Which of these methods for review shortcuts would you recommend (consider) when developing a Diagnostic RR? Why?
Are any of these methods unacceptable for a high quality RR? If so why?
Would you implement any of these methods for review shortcuts when developing a Diagnostic RR? Why/why not?

C. Parallelisation of tasks:

Those methods focus on increasing the intensity of work on review processes where multiple reviewers simultaneously complete review steps, for example, eligibility screening, data abstraction and risk-of-bias assessment.

Method	%
**Multiple reviewers completing the eligibility screening**	32
**Multiple reviewers completing the quality appraisal**	32
**Multiple reviewers completing the data abstraction**	28
**Multiple reviewers assessing the certainty of the evidence**	4
**Performing selected review activities simultaneously**	4

Key questions
Which of these methods for parallelisation of tasks would you recommend (consider) when developing a Diagnostic RR? Why?
Are any of these methods unacceptable for a high quality RR? If so why?
Would you implement any of these methods for parallelisation of tasks when developing a Diagnostic RR? Why/why not?

D. Automation:

Developing, adapting and using new technologies to fast-track the standard systematic review steps, for example, screening or data abstraction.

Method	%
**Used to assist in the screening/selection of references**	12
**Used to assist in the data abstraction**	8
**Used to assist in the quality appraisal**	4

Key questions
Which of these automation methods would you recommend (consider) when developing a Diagnostic RR? Why?
Are any of these methods unacceptable for a high quality RR? If so why?
Would you implement any of these automation methods when developing a Diagnostic RR? Why/why not?

E. Additional information

- Considering all of the methods used to speed up the review process in an RR, which methods do you think should definitely be used?

- Considering all of the methods used to speed up the review process in an RR, which methods do you think should definitely be avoided?

- Is there a method or approach that we have not talked about that you would like to mention?

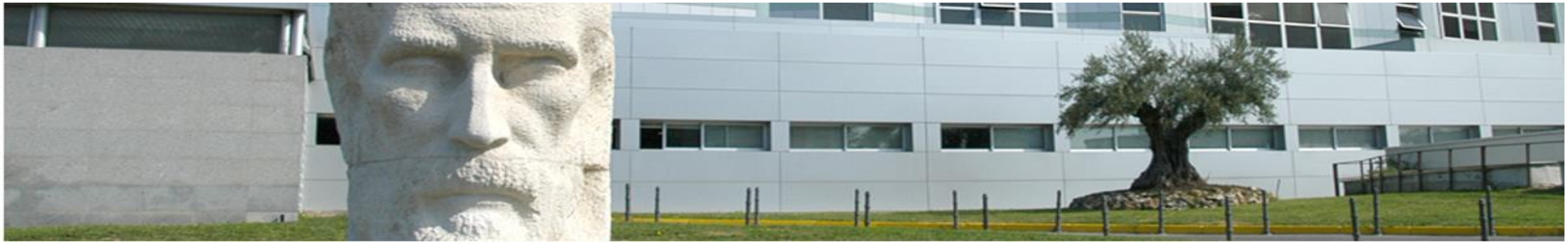

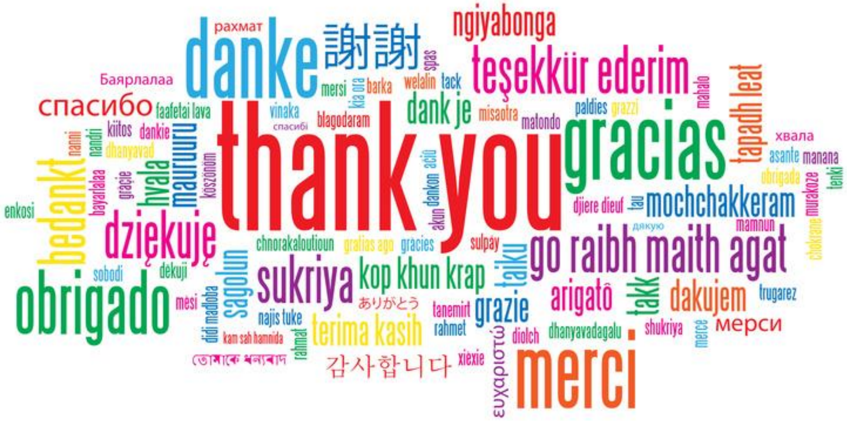
рахмат
danke
謝謝
ngiyabonga
teşekkür ederim
Баярлалаа
спасибо
dank je
thank you
gracias
tapadh leat
bedankt
dziękuję
go raibh maith agat
mochchakkeram
obrigado
sukriya
kop khun krap
arigatô
dakujem
grazie
terima kasih
merci
мерси
감사합니다
ありがとう
ευχαριστώ
